# Comprehensive analysis of mRNA and lncRNA expression for predicting lymph node metastasis in cervical cancer: a novel seven-gene signature approach

**DOI:** 10.3389/fgene.2025.1524821

**Published:** 2025-05-15

**Authors:** Jiahui Wei, Ming Wang, Yumei Wu

**Affiliations:** Department of Gynecologic Oncology, Beijing Obstetrics and Gynecology Hospital, Capital Medical University. Beijing Maternal and Child Healthcare Hospital, Beijing, China

**Keywords:** cervical cancer, lymph node metastasis, mRNA, lncRNA, prediction model

## Abstract

**Objective:**

Lymph node metastasis (LNM) critically determines recurrence and survival in cervical cancer (CC), yet current imaging-based methods lack accuracy. Retroperitoneal lymph node dissection leads to many adverse events. This study aimed to develop a clinically actionable molecular signature to predict LNM, enabling personalized surgical planning and improved patient outcomes.

**Methods:**

Transcriptome profiles and clinical data from 193 CC patients, encompassing information on LNM from The Cancer Genome Atlas (TCGA) and an external cohort (GSE26511), were analyzed. The differential expression of mRNAs and lncRNAs was identified using DESeq2. Subsequently, dual machine learning strategies—LASSO regression and the Boruta algorithm—were applied to select robust biomarkers. Finally, the seven-mRNA–lncRNA gene cluster was verified in tumor tissues of CC patients with and without LNM using qRT-PCR.

**Results:**

The seven-mRNA–lncRNA gene cluster included four mRNAs (ART3, HRG, MAPT, and SYTL5) and three lncRNAs (AC011239.1, AC125616.1, and RUVBL1.AS1). The expression patterns of the seven DEGs align with their levels in CC tissues. The signature demonstrated high predictive accuracy (AUC: 0.855 in training and 0.807 in testing cohorts). External validation using the GSE26511 dataset confirmed its clinical applicability (AUC: 0.611). Patients with high LNM scores exhibited poorer survival outcomes than those with low LNM scores (*p* = 0.0034).

**Conclusion:**

We constructed a reliable prediction model of LNM in CC patients with a seven-mRNA–lncRNA gene cluster. This model guides lymphadenectomy decisions, reduces overtreatment, and enhances patient survival. Our work bridges molecular insights with clinical practice and provides a foundation for further research into the management of CC.

## Introduction

Cervical cancer (CC) is a prevalent gynecological malignancy that poses significant risks to women’s health and overall wellbeing. It ranks as the fourth most common cancer diagnosed in women globally, with an estimated 660,000 new cases anticipated in 2022, representing 6.8% of all cancers in the female population. Additionally, this type of cancer is associated with a substantial mortality rate, accounting for 8.1% of all cancer-related deaths among women internationally (Francoeur et al., 2025). With the promotion of CC screening, the majority of CC cases are diagnosed at an early stage (Bruni et al., 2022; El-Zein and Franco, 2025). In patients with early-stage CC, the rates of lymph node metastasis (LNM) are approximately 12% ∼ 22%, 10% ∼ 27%, and 34% ∼ 43% in stage ⅠB, stage ⅡA, and stage ⅡB, respectively—the main factor associated with poor prognosis (Guo et al., 2023; Sakuragi, 2007). In the early and middle stages, the 5-year survival rate of CC without LNM is close to 90%, while the 5-year survival rate drops sharply to 65% in CC with LNM (Qin et al., 2024). Therefore, the accurate prediction of LNM is crucial in the management of this population.

There are two common methods to estimate LNM: surgical pathology and imaging. For most patients with early-stage CC at low LNM risk, unnecessary lymph node dissection tends to prolong surgical operation time, increase surgical complications, and reduce patients’ postoperative quality of life (Francoeur et al., 2025; Wang et al., 2022). Currently, the preoperative assessment of LNM in patients with CC is usually performed by imaging techniques such as CT, MRI, and PET-CT, but their accuracy in predicting lymph node invasion varies and remains a topic of debate. The reported overall sensitivity and specificity for CT are 51%–57% and 87%–91%, respectively; for PET-CT, they are 57%–66% and 95%–97%; for MRI, 54%–57% and 93%. These findings indicate a significant risk of missed diagnoses associated with these imaging techniques (Distelbrink et al., 2025). When combined with inflammation, tuberculosis, and lymph node proliferative lesions, it is more difficult to differentiate reactive lymph node enlargement from metastatic lymph node enlargement on tumors when imaging (Kido and Nakamoto, 2021). Therefore, it is urgent to find a new method to predict LNM to personalize treatment and improve the prognosis and survival rates of CC patients.

Long non-coding RNAs (lncRNAs) are known to play a complex and precise regulatory role in cancer development through the epigenetic regulation of mRNAs (Ying et al., 2023). Recent evidence increasingly supports the significant role of lncRNAs in the development and functioning of the lymphatic vasculature. Furthermore, recent studies underscore lncRNAs as key regulators of metastasis via epigenetic mechanisms (Ivanov et al., 2023). Guo et al. (2023) constructed TEKT2 and RPGR-based LNM-associated gene markers to predict prognosis and immune infiltration in CC patients. Tian et al. (2024) developed an eight-IRlncR-based prediction model that has the potential to be an important tool for predicting chemotherapeutic responses and prognosis for CC patients. Currently, there is no established predictive model that effectively combines transcriptome profiles and epigenetic regulation profiles to forecast LNM in CC patients. Therefore, we conducted a study to predict LNM based on the gene signature of transcription and epigenetics in cervical cancer. In this study, we present a comprehensive analysis of mRNA and lncRNA, employing machine learning techniques (LASSO and Boruta) to identify a minimal gene signature for predicting LNM in CC patients. The objectives of this model are to achieve high diagnostic accuracy, validate its effectiveness across multi-center cohorts, demonstrate robust performance capabilities, and correlate with survival outcomes—thereby bridging molecular discovery and clinical utility.

## Materials and methods

### Data collection

The transcriptome raw data of cervical cancer and related clinical information were downloaded from the TCGA database (https://portal.gdc.cancer.gov/), and cases with a clear histological diagnosis of cervical cancer and complete clinical information were filtered out. GTF files (GRCH38) were downloaded from the Encyclopedia of DNA Elements (https://www.gencodegenes.org/). Meanwhile, the original expression of each gene was log2 processed uniformly. We obtained 19,938 mRNAs and 16,882 lncRNAs associated with cervical cancer from the TCGA database.

### Differentially expressed genes

Differential expression analyses of lncRNAs and mRNAs of patients with and without LNM were performed using the R package “DESeq2.” Those lncRNAs and mRNAs with |log2 (fold change) | > 1 and false discovery rate (FDR)-adjusted *p* < 0.05 were identified as differentially expressed RNAs (Chen et al., 2020).

### Functional enrichment analysis

Gene Ontology (GO) and Kyoto Encyclopedia of Genes and Genomes (KEGG) analyses were performed through the clusterProfiler package to infer possible biological function. P*adj* <0.05 and gene number ≥2 were identified as associated with LNM. The LncSEA database (http://bio.liclab.net/LncSEA/index.php.) was used to enrich differentially expressed lncRNAs associated with LNM to further clarify the possible pathways associated with cervical cancer development (Chen et al., 2021).

### Screening of core genes associated with LNM and construction of the predictive model

A LASSO algorithm approach with five-fold cross-validation and a Boruta algorithm were used to select the differentially expressed mRNAs and lncRNAs, respectively, associated with LNM. The TCGA database was divided into a training and a testing cohort at a ratio of 2:1. In the training cohort, we constructed a logistic regression model using core RNAs for predicting LNM, which was validated in the testing cohort. GSE26511 from the GEO database (http://www.ncbi.nlm.nih.gov/geo) was used as an external validation database; it contains data from 39 cervical cancer patients with information on LNM. After that, we constructed a risk score formula for predicting LNM in cervical cancer using the weighted regression coefficients from the TCGA database training cohort. In this formula, “N” represents the number of core RNAs, “exp” represents the expression level of RNAs, and “coef” represents the regression coefficient of the corresponding RNAs in logistic regression analysis.

$$\text{RiskScore}=\sum_{i=1}^{N}\left(\exp*\text{coef}\right)$$

In order to verify whether the risk score is an independent predictor of LNM in CC, we conducted univariate and multivariate logistic regressions using the risk score with clinical variables.

### Risk score predicts survival in cervical cancer patients

The ROC curve was utilized to determine the optimal cutoff value for the risk score in predicting LNM. Based on this cutoff value, cervical cancer patients were categorized into high- and low-risk groups. Subsequently, the survival curves between the two groups were compared.

### Patients and tumor tissue samples

Tissues with pathologically confirmed cervical cancer were obtained from the sample database of the Beijing Gynecology and Obstetrics Hospital. All patients included in the study had not received any preoperative treatment and gave written informed consent.

### RNA extraction and quantitative real-time PCR analysis

A Total RNA Extraction Kit (Beijing Solarbio Technology, Beijing, China) was used to extract RNA from six patients’ tumor tissue samples. cDNAs were synthesized using the RevertAid RT kit (Thermo Fisher Scientific, Beijing, China). RT-PCR was performed using the SYBR green assay (Beijing Qihangxing Biotechnology, Beijing, China) on an AB 7500 machine (Applied Biosystems Inc., United States). The SYBR primers used in this study are listed in Supplementary Table S1. β-actin served as an internal control for normalization. The relative RNA abundance (fold change) of each mRNA and lncRNA in six patients’ tumor tissues was calculated using the standard 2^−ΔΔCT^. Each sample was examined in triplicate.

### Statistical analysis

All statistical analyses were performed using the statistical programming language R. The “glmnet” and Boruta packages implemented the LASSO and Boruta algorithms, respectively. The “pROC” and “ggsurvplot” packages plotted the ROC and Kaplan–Meier survival curves, respectively. GraphPad Prism V.8 was used for qRT-PCR analysis for graphing. In all analyses, *p* < 0.05 was used to indicate statistical difference.

## Results

### Characteristics of the study population

**FIGURE 1:**
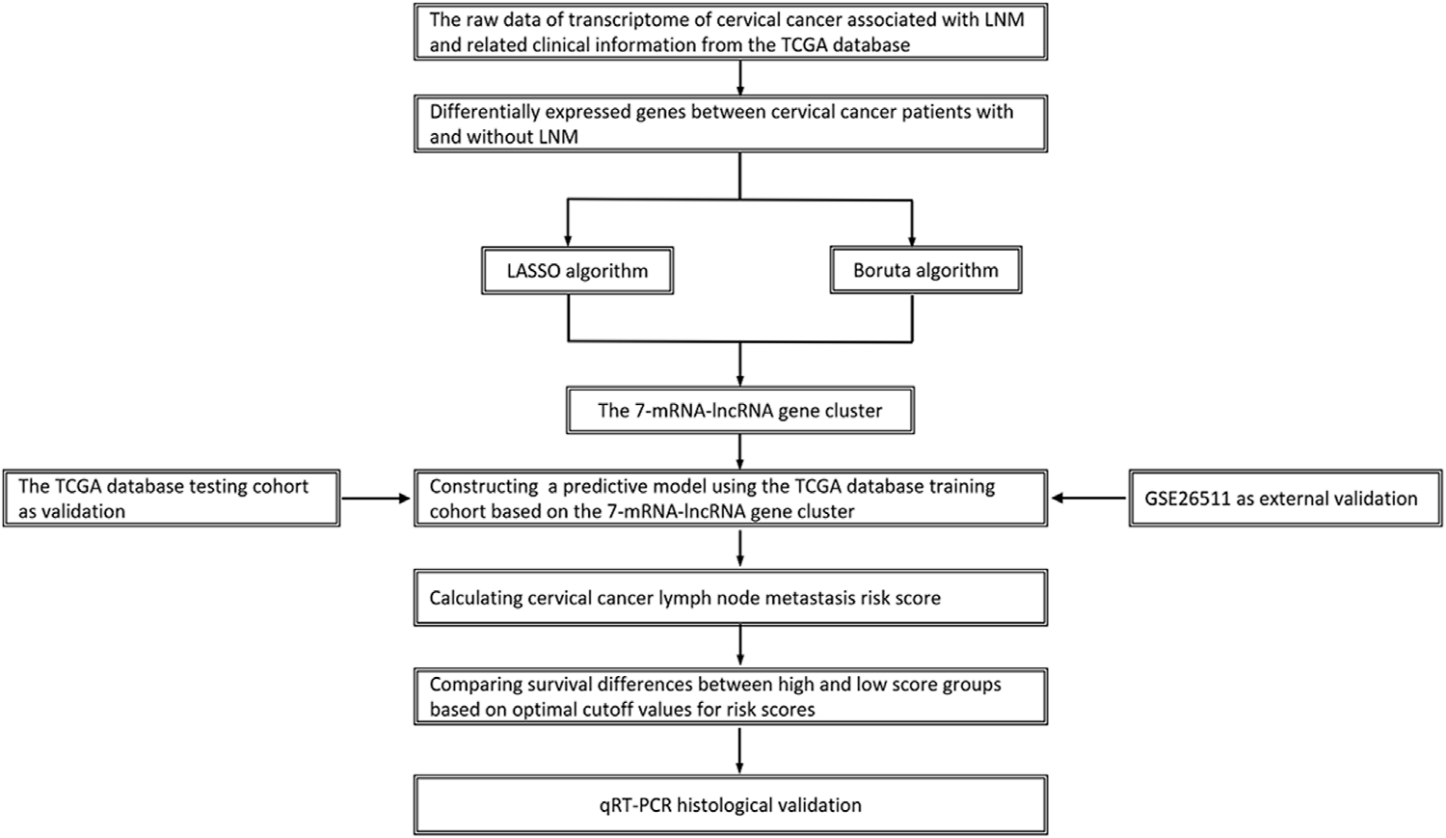
Flowchart of study design.

We confirmed 193 cervical cancer patients with intact information of LN status, including 133 (88.6%) without LNM and 60 (11.4%) with LNM (Table 1). Figure 1 shows a flowchart of our data collection, categorization, and analysis.

**TABLE 1:** Clinical characteristics of 193 cervical cancer patients from the TCGA database.

Variables	LNM (n = 60)	Non-LNM (n = 133)
Age (mean, years)	46	47
Gynecological stage		
I	31 (51.67)	99 (74.44)
II	8 (13.33)	29 (21.80)
III	19 (31.67)	3 (2.26)
IV	2 (3.33)	2 (1.50)
Grade		
Well differentiated	2 (3.33)	16 (12.03)
Moderately differentiated	29 (48.33)	61 (45.86)
Poorly differentiated	29 (48.33)	56 (42.11)
Histology type		
Squamous	51 (85.00)	105 (78.95)
Adenocarcinoma	6 (0.10)	19 (14.29)
Other	3 (0.05)	9 (6.77)

### Functional enrichment analysis of differentially expressed genes

**FIGURE 2:**
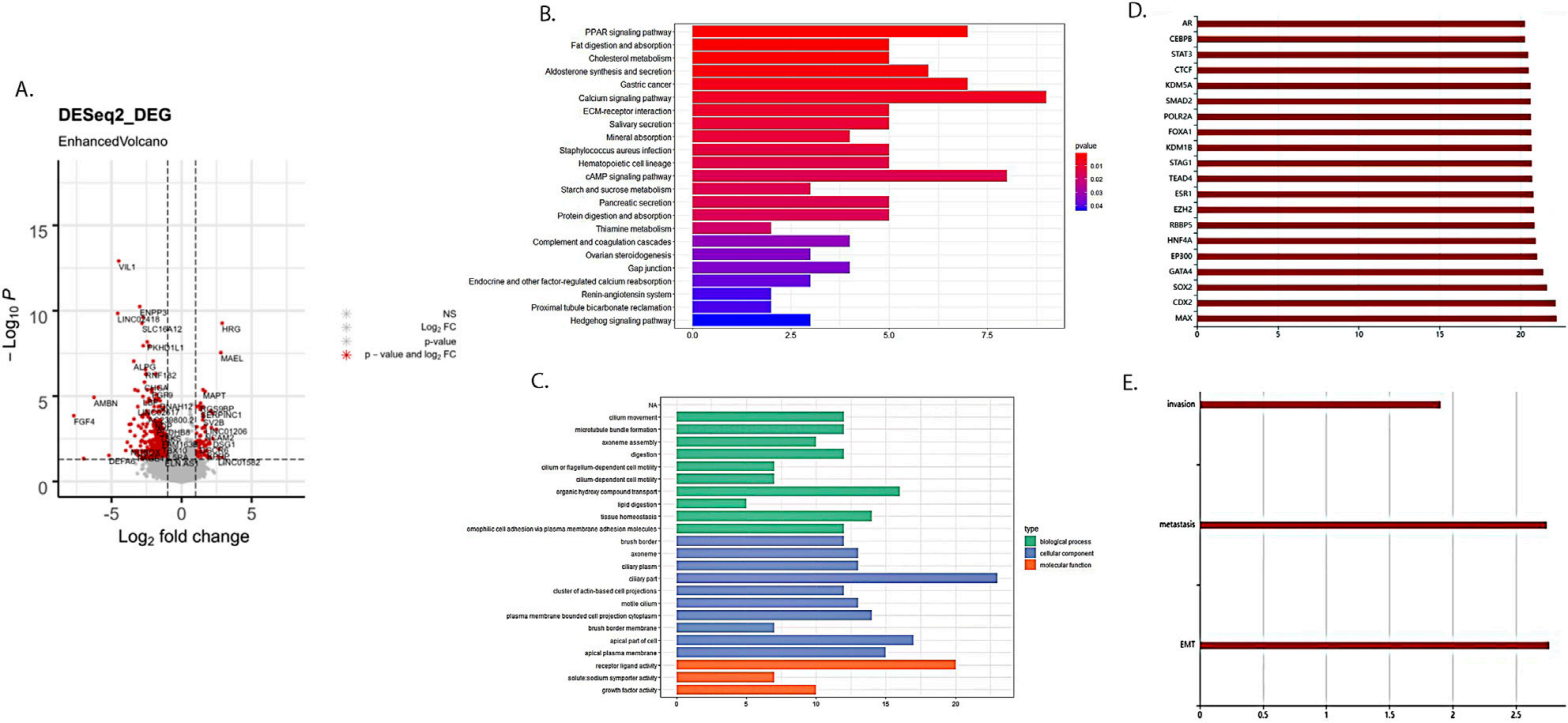
Differentially expressed RNAs between cervical cancer patients with and without LNM. **(A)** Volcano plot of differential RNAs. Red dots represented differentially expressed RNA. **(B)** KEGG enrichment analysis of differentially expressed mRNAs related to cervical cancer LNM. **(C)** GO enrichment analysis of differentially expressed mRNAs related to cervical cancer LNM. **(D)** Enrichment analysis results of differentially expressed lncRNAs related to cervical cancer LNM on binding proteins. **(E)** Enrichment analysis results of differentially expressed lncRNAs related to cervical cancer LNM in cancer characteristics.

We obtained 443 differentially expressed mRNAs and lncRNAs (358 mRNAs and 85 lncRNAs), including 372 mRNAs and lncRNAs that were significantly downregulated and 71 mRNAs and lncRNAs significantly upregulated (Figure 2A).

The KEGG enrichment analysis revealed that differentially expressed mRNAs were involved in various aspects of tumor development, such as the PARP signaling pathway, lipid metabolism, and the cAMP signaling pathway (Figure 2B). The GO pathway revealed that differentially expressed mRNAs were involved in the regulation of tumor progression in terms of cell cycle, tissue homeostasis, cellular adhesion, and so on (Figure 2C). Meanwhile, lncRNAs mainly focused on binding proteins such as STAT3 and CTCF, as well as cancer features such as invasion, metastasis, and epithelial–mesenchymal transition (Figures 2D,E).

### Identification of mRNAs and lncRNAs associated with LNM in cervical cancer

**FIGURE 3:**
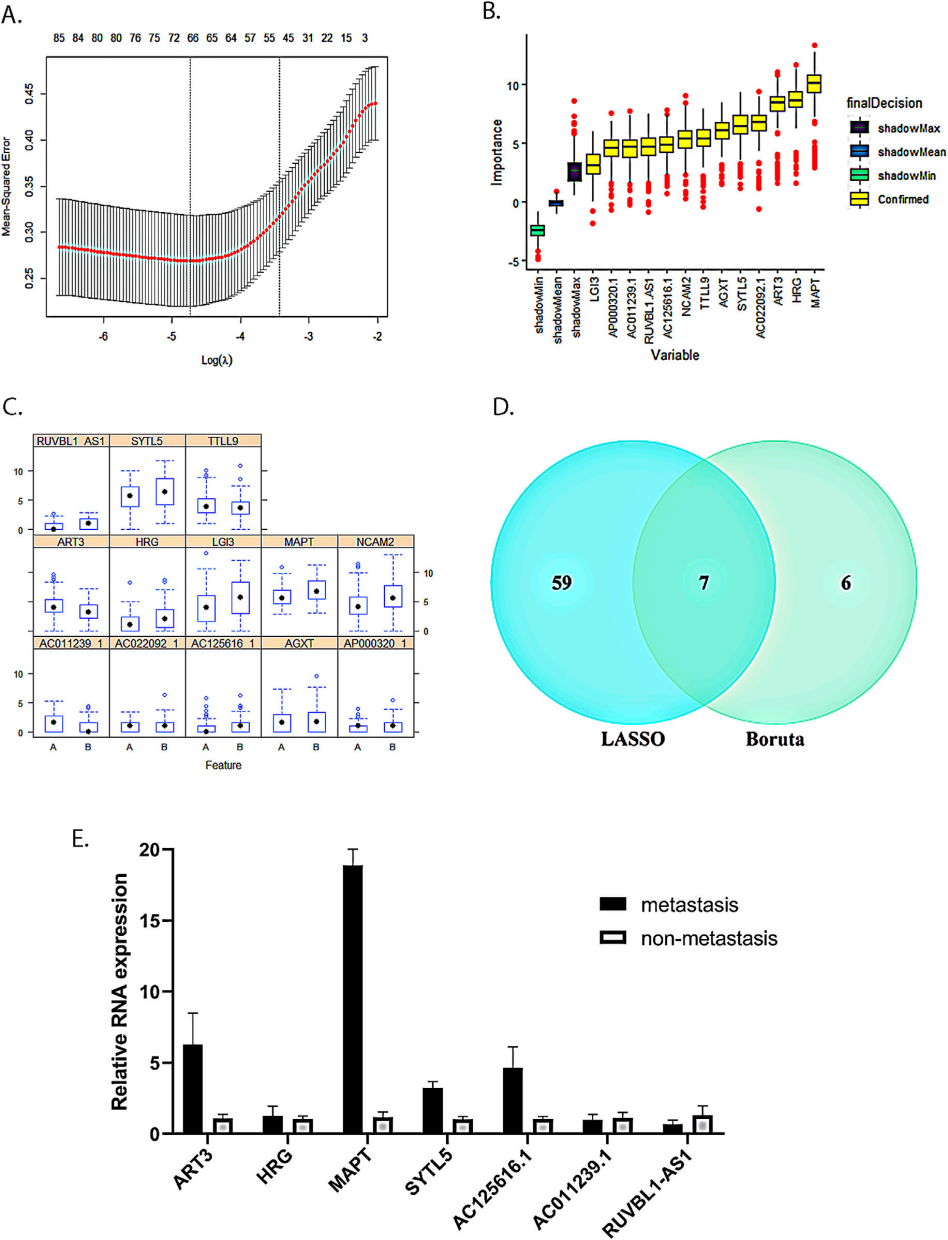
Seven-mRNA–lncRNA gene cluster screening. **(A)** LASSO algorithm with five-fold cross-validation used to screen cervical cancer LNM-related mRNAs and lncRNAs. Through cross-validation, we found that the number of variables corresponding to λ under the minimum error was 66 mRNAs and lncRNAs. **(B)** Screening of cervical cancer LNM-related mRNAs and lncRNAs using the Boruta algorithm. **(C)** Expression distribution of 13 key RNAs screened by the Boruta algorithm in two groups with and without LNM. A is the group without LNM, and B is the LNM group. **(D)** The intersection of the LASSO algorithm and the Boruta algorithm results. **(E)** Verification of the seven-mRNA–lncRNA gene cluster in cervical cancer using qRT-PCR.

We used the LASSO algorithm to screen the differentially expressed genes and identified 66 key RNAs (43 mRNAs and 23 lncRNAs, Figure 3A). Meanwhile, we employed the Boruta algorithm to identify 13 key RNAs (8 mRNAs and 5 lncRNAs, Figure 3B) among the differentially expressed genes. We also examined the expression patterns of these 13 key RNAs in both the LNM and the non-LNM CC groups (Figure 3C). We then took the intersection of the LASSO algorithm and the Boruta algorithm results as the final core RNA omics feature for this study (Figure 3D). Eventually, we obtained a seven-mRNA–lncRNA gene cluster (four mRNAs: ART3, HRG, MAPT, and SYTL5; three lncRNAs: AC011239.1, AC125616.1, and RUVBL1.AS1) that was mostly relevant to LNM of cervical cancer. Among these transcripts, AC125616.1, HRG, MAPT, RUVBL1.AS1, and SYTL5 were found to be upregulated, while AC011239.1 and ART3 were downregulated in cervical cancer patients with LNM compared to those without it.

### Expression of the seven-mRNA–lncRNA gene cluster in tumor tissues of patients with LNM from cervical cancer

To further investigate the relationship between the expression of the seven-mRNA–lncRNA gene cluster and LNM, we conducted qRT-PCR analysis to measure the expression levels of the gene cluster in cervical cancer patients with LNM and those without it. Our results revealed that HRG, MAPT, SYTL5, and AC125616.1 were upregulated in the primary tumors of the LNM group compared to the non-LNM group, which is consistent with our model results. On the other hand, AC011239.1 showed downregulated expression in the LNM group, also in accordance with the model results. However, ART3 exhibited upregulated expression and RUVBL1.AS1 showed downregulated expression in the LNM group, which were not consistent with the model results. These discrepancies might be attributed to the limited number of samples in our study introducing a potential bias (Figure 3E).

### Construction of a predictive model for LNM in cervical cancer using the 7-mRNA-lncRNA gene cluster

**FIGURE 4:**
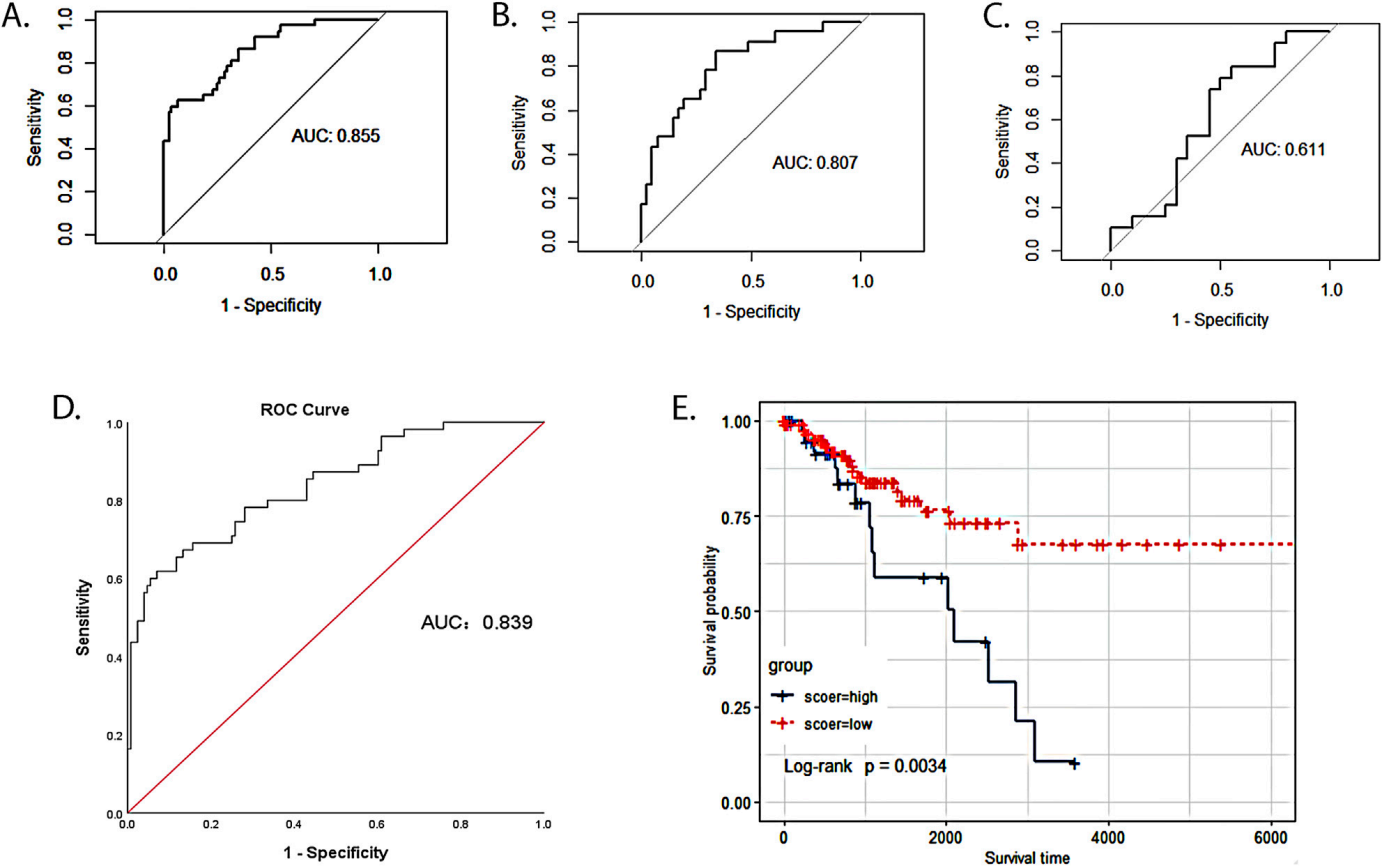
Value of the seven-mRNA–lncRNA gene cluster in predicting LNM of cervical cancer. **(A)** ROC curve of seven-mRNA–lncRNA gene cluster in predicting LNM in the TCGA training cohort (the value of AUC: 0.855, 95%CI: 0.784–0.927). **(B)** ROC curve of seven-mRNA–lncRNA gene cluster in predicting LNM in the TCGA training cohort (value of AUC: 0.807, 95%CI: 0.700–0.918). **(C)** ROC curve of seven-mRNA–lncRNA gene cluster in predicting LNM in GSE26511 (value of AUC: 0.611, 95%CI: 0.427–0.794). **(D)** ROC curve of seven-mRNA–lncRNA gene cluster risk score in predicting LNM of cervical cancer (optimal cutoff value:1.1, value of AUC: 0.839, 95%CI: 0.775–0.904). **(E)** The survival time of the low-risk group was compared with that of the high-risk group to examine the relationship between the seven-mRNA–lncRNA gene cluster and the prognosis of cervical cancer patients. The blue curve represented the high-risk group with a high score, and the red curve represented the low-risk group with a low score (p = 0.0034).

We divided 193 cervical cancer patients from the TCGA database into a training cohort (n = 129; 85 patients without LNM and 44 patients with LNM) and a testing cohort (n = 64; 48 patients without LNM and 16 patients with LNM) according to a 2:1 ratio. In the training cohort, we developed a logistic regression prediction model based on the seven-mRNA–lncRNA gene cluster core features. ROC analysis revealed that the seven-mRNA–lncRNA gene cluster achieved an AUC value of 0.855 (0.784–0.927) (Figure 4A).

### Independent validation of the seven-mRNA–lncRNA gene cluster in different cohorts

To examine the robustness and reproducibility of the seven-mRNA–lncRNA gene cluster for predicting LNM, we further tested it in two other patient cohorts. In the testing cohort, ROC analysis revealed that the seven-mRNA–lncRNA gene cluster achieved an AUC value of 0.807 (0.700–0.918) (Figure 4B). In GSE26511, an external validation cohort, the seven-mRNA–lncRNA gene cluster achieved an AUC value of 0.611 (0.427–0.794) (Figure 4C).

### Risk score constructed from the seven-mRNA–lncRNA gene cluster for LNM in cervical cancer

We utilized the logistic regression model from the training cohort to assign weights to the coefficients of the seven-mRNA–lncRNA core features. These weights were then used to calculate a risk score through a linear combination.

$$\begin{array}{c} \text{RiskScore}=\left(-0.185*\text{EAC}011239.1\right)+\left(0.0173*\text{EAC}125616.1\right)+\left(-0.0248*\text{EART}3\right)\\ +\left(0.028*\text{EHRG}\right)+\left(0.0224*\text{EMAPT}\right)+\left(0.3047*\text{ERUVBL}1-\text{AS}1\right)+\left(0.0021*\text{ESYTL}5\right) \end{array}$$

To determine whether the predictive ability of the seven-mRNA–lncRNA gene cluster for LNM in cervical cancer was independent of other clinical features, we conducted logistic regression analysis. The results of univariate logistic regression in the training cohort indicated a significant association between the risk score and LNM. Higher risk scores were associated with a greater likelihood of LNM in cervical cancer (high-risk group vs low-risk group: OR = 3.248, 95% CI = 1.873–4.869, *p* < 0.001, n = 129, Table 2). Multivariate logistic regression in the training cohort also showed a significant association between the risk score and LNM (high-risk group vs low-risk group: OR = 3.580, 95% CI = 2.099–5.309, *p* < 0.001, n = 64, Table 3). We also performed the same analysis in the testing cohort and obtained similar results (Table 4, 5). These findings suggested that the seven-mRNA–lncRNA gene cluster can be considered an independent risk factor for predicting LNM in cervical cancer.

**TABLE 2:** Univariate logistic regression analysis for predicting cervical cancer lymph node metastasis in TCGA training cohort.

Variables	OR value	95% confidence interval		P
		Upper limit	Lower limit	
Age	−0.007	−0.038	0.022	0.627
Gynecological stage	0.966	0.479	1.494	<0.001
Grade	0.743	−0.346	0.860	0.426
Histology type	−0.418	−1.260	0.273	0.274
Risk score	3.248	1.873	4.869	<0.001

**TABLE 3:** Multivariate logistic regression analysis for predicting cervical cancer lymph node metastasis in TCGA training cohort.

Variables	OR value	95% confidence interval		P
		Upper limit	Lower limit	
Age	−0.005	−0.042	0.032	0.804
Gynecological stage	1.143	0.595	1.744	<0.001
Grade	0.162	−0.580	0.926	0.671
Histology type	0.093	−0.871	0.966	0.839
Risk score	3.580	2.099	5.309	<0.001

**TABLE 4:** Univariate logistic regression analysis for predicting cervical cancer lymph node metastasis in TCGA testing cohort.

Variables	OR value	95% confidence interval		P
		Upper limit	Lower limit	
Age	−0.012	−0.055	0.029	0.574
Gynecological stage	0.865	0.136	1.072	0.022
Grade	0.617	−0.224	1.533	0.165
Histology type	0.086	−1.000	1.079	0.866
Risk score	3.053	1.398	5.090	<0.001

**TABLE 5:** Multivariate logistic regression analysis for predicting cervical cancer lymph node metastasis in TCGA testing cohort.

Variables	OR value	95% confidence interval		P
		Upper limit	Lower limit	
Age	−0.041	−0.096	0.009	0.118
Gynecological stage	1.327	0.378	2.452	0.011
Grade	0.701	−0.407	1.938	0.234
Histology type	0.711	−0.500	1.960	0.239
Risk score	3.776	1.734	6.411	0.001

### The seven-mRNA–lncRNA gene cluster as a prognostic biomarker for cervical cancer

After removing ten cervical cancer samples with no survival time or status, we calculated the optimal cutoff value of 1.1 for the seven-mRNA–lncRNA gene cluster to predict the risk score of LNM in cervical cancer using the ROC curve, and the AUC value was 0.839 (95% CI = 0.775–0.904) (Figure 4D). In this study, 183 cervical cancer patients with LN information from the TCGA database were categorized into low- (n = 140) and high-risk (n = 43) groups based on the optimal cutoff value of the risk score. The survival time of the low-risk group was compared with that of the high-risk group to examine the relationship between the seven-mRNA–lncRNA gene cluster and the prognosis of cervical cancer patients. The survival curves revealed a significantly lower survival rate in the high-risk than the low-risk group (*p* = 0.0034, Figure 4E), indicating the reliability of the seven-mRNA–lncRNA gene cluster in predicting the prognosis of cervical cancer patients.

## Discussion

The metastasis of lymph nodes in CC is critical for determining patient prognosis and guiding treatment selection (Guo et al., 2025). Preoperative imaging assessments of lymph nodes often yield a high rate of missed diagnoses, while unnecessary surgical lymph node dissections increase patient burden and risk complications (Dheur et al., 2024). Unlike previous studies that focused solely on individual mRNA or lncRNA for CC prognosis, this study integrated both mRNA and lncRNA by employing two machine-learning algorithms (LASSO and Boruta). It identified a seven-gene cluster comprising both mRNA and lncRNA associated with LNM in CC. Furthermore, we constructed a predictive model based on this gene cluster. Compared to earlier models, ours demonstrated superior performance in predicting LNM in CC, achieving an AUC of 0.855 in the training cohort and 0.807 in the testing cohort, with validation through independent external datasets. Moreover, patients with higher risk scores in this model exhibited worse prognoses compared to those with lower risk scores, providing a theoretical foundation for targeted treatment and management in this population. Our model aids in the reduction of unnecessary lymph node dissection, facilitating early detection and assisting in the selection of adjuvant treatment plans, thereby enabling personalized medical care.

RNAs serve as a medium for the communication and delivery of genetic information, enabling the expression of genetic information at the protein level (Ivanova et al., 2023). In the context of cervical cancer, several genetic markers have been developed to predict prognosis and recurrence, thereby assisting in clinical decision-making (Lizano et al., 2024). Some RNAs in the seven-mRNA–lncRNA gene cluster have been reported to be associated with cancer development and progression. ART3 influences cell proliferation and metastasis by modulating the Akt/ERK signaling pathway across various tumors (Zhong et al., 2020). Zhang et al. (2020) developed a coding–noncoding signature related to LNM in CC and validated that ART3 expression was downregulated in LNM patients—aligning with our findings. We observed that ART3 expression was downregulated in the LNM group, which may enhance the invasiveness of tumor cells by impairing DNA repair mechanisms or promoting genomic instability. Proteins encoded by the HRG gene serve as crucial targets for the proliferation, migration, and formation of endothelial cells within specific tumor microenvironments (Morrissey, 2024; Pan et al., 2022). This study demonstrated that HRG expression is upregulated in CC with LNM, indicating its association with tumor progression and metastasis. Its upregulation may promote lymphangiogenesis, thereby providing a pathway for tumor cells to spread to lymph nodes. Callari et al. (2023) conducted a computerized pan-cancer analysis of MAPT transcriptome profiling, revealing that MAPT expression is associated with various cancer characteristics, including inflammation, proliferation, and epithelial-to-mesenchymal transition. The increased expression of MAPT is correlated with a poor prognosis and resistance to paclitaxel-based chemotherapy in cancer patients (Lin et al., 2024). Our study found that MAPT was upregulated in CC patients with LNM, indicating that these patients may have a poorer prognosis and a higher likelihood of developing resistance to paclitaxel treatment. Its elevated expression may facilitate lymph node colonization by enhancing cell migration capabilities or by inhibiting apoptosis. SYTL5, a positive regulator of chemotactic leukocyte migration, plays a crucial role in the development and modulation of immune and inflammatory responses (Colvin et al., 2010). The upregulation of SYTL5 promotes tumor progression and exerts an oncogenic effect by activating the NF-κB signaling pathway (Huang et al., 2022). In this study, we found that SYTL5 was upregulated and may play an oncogenic role in CC. The upregulation of SYTL5 may induce the secretion of pro-inflammatory factors, recruit immunosuppressive cells, and contribute to the formation of a pre-metastatic microenvironment. Additionally, RUVBL1.AS1 may influence the expression of metastasis-related genes through epigenetic regulation, including chromatin remodeling (Bi et al., 2025).

The predictive performance of the seven-mRNA–lncRNA gene cluster in predicting LNM in cervical cancer has been validated across multiple patient cohorts, demonstrating good performance. However, this study has certain limitations. First, while we emphasized predictive accuracy, we did not explore the biological mechanisms underlying the identified mRNAs and lncRNAs associated with LNM. We will pursue deeper exploration and verification in future research, focusing on validating the biological basis and functions of these mRNAs and lncRNAs in predicting LNM in cervical cancer through basic functional mechanism experiments. Second, due to the small sample size in certain subgroups of TCGA data, effective stratified analysis might not be feasible. We aim to validate the applicability of the model in various subgroups using larger samples in the future and to develop a multidimensional prediction tool that integrates molecular markers and clinical features. Finally, although the model reduced the risk of overfitting through multiple validation strategies, the simplified feature set of seven genes might not fully encompass the molecular heterogeneity of cervical cancer LNM. Future studies should aim to expand the sample size to optimize the model, integrate multi-omics data (such as methylation and protein interaction networks) to enhance the biological depth of the model, and explore its generalizability across different populations.

Lymphadenectomy may result in severe pelvic adhesions, which can further lead to infertility or ectopic pregnancy in young patients. Additionally, lymphadectomy is necessary to assess the possibility of ongoing pregnancy in pregnant patients diagnosed with early-stage CC. Furthermore, elderly patients may not tolerate the adverse events caused by lymphadenectomy. The prediction model for LNM developed in our study may be an adjuvant tool in decision-making about lymphadenectomy during CC surgery in the above population. The gene cluster identified in this study may also provide a molecular target to reduce the risk of LNM or distant metastasis, such as mRNA vaccination.

## Conclusion

This study integrated a seven-gene signature comprising mRNA and lncRNA and, using machine learning and multi-cohort validation, achieved prediction of LNM in CC. This advancement can guide decision-making regarding lymphadenectomy, thereby reducing the risk of overtreatment. Furthermore, it provides a theoretical basis for targeted treatment and management from a molecular perspective. In the future, further validation will be conducted through large-sample, multi-omics studies, which will inform clinical diagnosis and treatment.

## Data Availability

The datasets presented in this study can be found in online repositories. The names of the repository/repositories and accession number(s) can be found in the article/Supplementary Material.
