# Supplementary material for: Comprehensive analysis of mRNA and lncRNA expression for predicting lymph node metastasis in cervical cancer: a novel seven-gene signature approach
Source: Front Genet. 2025 May 15;16:1524821. doi: 10.3389/fgene.2025.1524821 (PMC12119550; doi:10.3389/fgene.2025.1524821)
Supplement: Supplementary file 1 [file Table1.docx]

Supplementary table 1 the 7-mRNA-lncRNA gene cluster SYBR primer comparison table

| RNA name | RNA Properties | Primer Sequence |
| --- | --- | --- |
| ART | mRNA | 5′-GCCTTGGAGTTGACATTGAA-3′ 5′-GGTTCTCAATACAGTTTTCGGTT-3′ |
| HRG | mRNA | 5′-GATCATCATCATCCCCACAAG-3′  5′-GGGTCACAAGGTCCATAGTC-3′ |
| MAPT | mRNA | 5ʹ-AAGATCGGCTCCACTGAGAA-3ʹ  5ʹ-ATGAGCCACACTTGGAGGTC-3ʹ |
| SYTL5 | mRNA | 5ʹ-GCCCCAATGGCAGCTG-3ʹ  5ʹ-TTAGCTGCGCGATTTTGTCAC-3ʹ |
| AC011239.1 | lncRNA | 5ʹ-AACGCTGGTGGTGTTTAGGC-3ʹ  5ʹ-AATCAAAGTTTGAAGGCAGCC-3ʹ |
| AC125616.1 | lncRNA | 5ʹ-GGATGCTAAAGAGCCAACCGA-3ʹ  5ʹ-CCTCACTGGTTTGTTCTGTCCT-3ʹ |
| RUVBL1.AS1 | lncRNA | 5ʹ-GTTCGGACTTCGGGTCAGTC-3ʹ  5ʹ-AGAGGGAAACAGACCATGCC-3ʹ |
| ACTIN | mRNA | 5ʹ-CCTCG CCTTT GCCGA TCC-3ʹ  5ʹ-GGATCTTCATGAGGTAGTCAGTC-3ʹ |
